# Using molecular network analysis to explore the characteristics of HIV-1 transmission in a China-Myanmar border area

**DOI:** 10.1371/journal.pone.0268143

**Published:** 2022-05-06

**Authors:** Yuying Zhang, Jie Dai, Zhengxu Li, Yanling Ma, Huichao Chen, Lijuan Dong, Xiaomei Jin, Min Yang, Zhijun Zeng, Pengyan Sun, Anyan Hu, Min Chen

**Affiliations:** 1 School of Public Health, Kunming Medical University, Kunming, Yunnan, China; 2 Institute for AIDS/STD Control and Prevention, Yunnan Center for Disease Control and Prevention, Kunming, Yunnan, China; 3 Division for AIDS/STD Control and Prevention, Baoshan Center for Disease Control and Prevention, Baoshan, Yunnan, China; Hebei Provincial Center for Disease Control and Prevention, CHINA

## Abstract

**Background:**

The China-Myanmar border area is considered a hot spot of active HIV-1 recombination in Southeast Asia. To better understand the characteristics of HIV-1 transmission in this area, a cross-sectional HIV-1 molecular epidemiological survey was conducted in Baoshan Prefecture of Yunnan Province.

**Methods:**

In total, 708 newly reported HIV-1 cases in Baoshan Prefecture from 2019 to 2020 were included in this study. HIV-1 *gag*, *pol* and *env* genes were sequenced, and the spatial and demographic distributions of HIV-1 genotypes were analyzed. The characteristics of HIV-1 transmission were investigated using the HIV-1 molecular network method.

**Results:**

In the 497 samples with genotyping results, 19 HIV-1 genotypes were found, with URFs being the predominant strains (30.2%, 150/497). The main circulating HIV-1 strains were mostly distributed in the northern area of Baoshan. URFs were more likely identified in Burmese individuals, intravenous drug users and those younger than 50 years old. CRF08_BC was more likely detected in farmers and those of Han ethnicity, CRF01_AE in the young and those of Han ethnicity, and CRF07_BC in the subpopulation with junior middle school education and higher. Moreover, CRF118_BC and CRF64_BC were more likely found in the subpopulation aged ≥40 years and ≥50 years, respectively. Among 480 individuals with *pol* sequence detection, 179 (37.3%) were grouped into 78 clusters, with Baoshan natives being more likely to be in the network. The proportion of the linked individuals showed significant differences when stratified by the regional origin, marital status, age and county of case reporting. In the molecular network, recent infections were more likely to occur among nonfarmers and individuals aged below 30 years.

**Conclusions:**

HIV-1 genetics has become complex in Baoshan. HIV-1 molecular network analysis provided transmission characteristics in the local area, and these findings provided information to prioritize transmission-reduction interventions.

## Introduction

Epidemiological investigation is the most direct and effective way to track transmission and identify transmission clusters, representing recent and ongoing HIV transmission. Key characteristics of the underlying risk can be identified based on transmission clusters, which helps to guide intervention efforts for ongoing transmission. Nevertheless, due to limited resources, privacy concerns and discrimination, epidemiological investigations face many obstacles. HIV molecular clusters are constructed based on genetic similarity among persons closely related by transmission, which represents a subset of the underlying transmission network [1]. Thus, the construction of molecular clusters provides a means for referring to transmission clusters. With the development of HIV molecular epidemiology, the application of molecular networks in AIDS prevention is gradually attracting attention [2, 3]. The HIV-1 molecular network has been used to explore factors related to HIV transmission in different regions and populations [4–6]. Moreover, the analysis methods of molecular networks have been improved and further combined with phylogenetic analysis to investigate the epidemiological characteristics, such as the judgment of new transmission events, the calculation of propagation frequency and the prediction of high-risk networks [7–11]. All of these aspects promote the advance and application of HIV molecular surveillance, which facilitates rapid detection and response to emerging clusters of HIV infection to further reduce transmission. Thus, HIV-1 molecular surveillance is one pillar of the Ending the HIV Epidemic plan in the United States [12].

Yunnan is located on the southwest border of China, bordering Myanmar, Laos and Vietnam. Because of its location along the drug trafficking routes from the “Golden Triangle” into China, Yunnan became the first region in China where the HIV-1 epidemic was detected among intravenous drug users (IDUs) [13]. In the last 30 years, Yunnan has been an areas in China heavily affected by HIV. By the end of 2018, more than 100,000 people were living with HIV/AIDS in Yunnan, ranking second in the nation. Retrospective studies have suggested that Yunnan was an important entry for HIV-1 strains into China in the early stage, including subtype B and subtype C in IDUs and CRF01_AE in female sexual workers (FSWs) [14–16]. Among IDUs, subtype B and subtype C frequently recombined, consequently diverse BC recombinant forms have originated and circulated, including CRF07_BC and CRF08_BC, which were identified in the late 1990s and spread to other parts of China through drug trafficking [17, 18]. Furthermore, some novel BC circulating recombinant forms (CRFs) were identified in western Yunnan in the last 10 years [19–23], and the proportion of unique recombinant forms (URFs) remains high in recent infections [24]. However, previous HIV molecular epidemiological studies have not revealed the characteristics of HIV transmission, though HIV molecular network analysis is a new tool toward this end.

Baoshan Prefecture is located in western Yunnan Province and borders Myanmar in the northwest (Tengchong County) and the south (Longling County). In the west, it is adjacent to Dehong Prefecture, where the first HIV-1 epidemic among IDUs was identified in China. Because of its special geographical location, Baoshan is also a key area for AIDS prevention and control in Yunnan Province. In particular, the high-risk transnational floating population might drive HIV transmission in border regions [25]. In 1991, the first HIV-1-infected individual was reported in Tengchong County. By the end of 2018, the proportion of diagnosed people living with HIV/AIDS (PLWHA) in Baoshan was estimated to be 79.3%. To effectively control the HIV epidemic, HIV testing and intervention need to be improved based on an accurate understanding of the characteristics of transmission. Due to long-term circulation, HIV-1 genetics have become complicated in Baoshan, where some novel CRFs have been identified [20, 21]. To lay a foundation for the establishment of an HIV molecular surveillance system and comprehensively understand the characteristics of HIV-1 transmission in the border area, we performed an HIV-1 molecular network study in Baoshan.

## Materials and methods

### Study participants and sample collection

From January 2019 to December 2020, 708 HIV-1-infected individuals were recruited at health care facilities that conduct HIV testing in Baoshan City, Yunnan Province, China, including local voluntary counseling and testing sites (VCTs), medical institutions and surveillance sites. The inclusion criteria were as follows: (1) aged 15 years and above; (2) newly reported HIV-1 cases; (3) currently living in Baoshan Prefecture; and (4) agreed to participate in the survey and signed an informed consent form. Blood samples and the relevant demographic information were collected. The participants were recruited consecutively. Among the newly reported cases aged 15 years and above during 2019–2020, 676 individuals were Chinese and 169 Burmese. Ultimately, 606 Chinese and 102 Burmese individuals were recruited. The reasons for the dropout were as follows: informed consent was not obtained, the cases could not be followed up, and blood samples were not collected. Consent was provided by adults or the guardians of the minors. This study was approved by the Biomedical Ethics Review Committee of Yunnan Center Disease Control and Prevention.

### Amplification and sequencing of HIV-1 gene fragments

HIV-1 RNA was extracted from 140 μl of plasma using a QIAamp Viral RNA Mini kit (Qiagen, Germany) following the manufacturer’s instructions. The *gag* (HXB2: 781–1861), *pol* (HXB2: 2147–3462) and *env* (HXB2: 7002–7541) genes were amplified using nested polymerase chain reactions (PCRs). The first round of PCR was performed with a One Step RNA PCR Kit (Takara, Dalian, China) in a 25 μl reaction volume, and the second round was performed with 2× Taq PCR MasterMix (Tiangen, Beijing, China) in a 50 μl reaction volume. The primers and procedures used for nested PCR and the primers used for sequencing are shown in S1 Table.

### Sequence analysis

The contigs obtained were assembled using Sequencher 5.1 (Gene Codes, Ann Arbor, MI). The assembled sequences were aligned with Bio-Edit 7.0 software and manually edited. HIV-1 reference sequences were selected and downloaded from the HIV databases of the Los Alamos National Laboratory (LANL) (http://hiv-web.lanl.gov). Neighbor-joining phylogenetic trees were construed with the Kimura 2-parameter model with 1000 bootstrap replicates in MEGA X. Sequences with possible intersubtype recombination were analyzed with the Recombination Identification Program (RIP, version 3.0; http://hiv-web.lanl.gov). HIV-1 genotyping was based on at least two segments of the *gag*, *pol* and *env* genes.

### Spatial distribution analysis of HIV-1 genotypes

The distribution density of each HIV-1 genotype was displayed using a dot density map, in which the percentage of a certain HIV-1 genotype in each county was shown as a corresponding number of dots; one dot was defined as 0.025% of the total cases with genotyping. The dot density map was prepared using Quantum GIS 3.16.

### Construction and analysis of HIV-1 molecular network

According to a previous study [26], an HIV-1 molecular network was constructed using phylogenetic analysis combined with genetic distance. The maximum likelihood (ML) phylogenetic tree was constructed with 480 *pol* sequences using FastTree software. Transmission clusters were extracted using Cluster Picker software. We evaluated the effect of different genetic distances on cluster identification according to a described method [27]. The node support threshold was ≥90%, and pairwise genetic distances varied from 0.5% to 5.0%, with an interval of 0.5%. As shown in S1 Fig, the number of clusters was nearly saturated beyond the genetic distance of 3.0%; that is, the proportion of increasing clusters decreased significantly. Thus, a genetic distance of 3.0% was selected. This cutoff value was also used in previous studies [26, 28]. In each cluster, nodes were linked according to the minimum genetic distances algorithm. The HIV-1 molecular network was visualized using Gaphi 0.9.2.

### Bayesian MCMC evolutionary analyses

The time of the most recent common ancestor (tMRCA) of clades within HIV-1 molecular clusters was estimated by Bayesian MCMC analysis for *pol* sequences using the BEAST package [29]. The analysis was performed using an uncorrelated exponential relaxed molecular clock method with ‘Bayesian Skyline’ coalescent tree priors under the general time reversible (GTR) nucleotide substitution model and a gamma (Γ4) plus invariant site heterogeneity model. Each MCMC analysis was run for at least 300 million generations and sampled every 30,000 generations. With a burnin of the initial 10% of trees from the output of MCMC analysis, the maximum clade credibility (MCC) tree was generated by TreeAnnotator v1.8.2. The MCC tree was visualized using FigTree V1.4.0, by which the tMRCAs of the clades were obtained.

### Sequence data

All of the sequences obtained in this study were submitted to GenBank under accession numbers OM453954-OM455370.

### Statistical analysis

Statistical analyses were processed using the SPSS 19.0 statistical analysis software package (SPSS Inc. Chicago, IL). Categorical variables were compared using the χ^2^ test. Univariate logistic analysis was first performed, and variables with p<0.10 were selected for multivariate logistic analysis. All tests were two-tailed and a p value <0.05 was considered statistically significant.

## Results

### Demographic characteristics of the study participants

From January 2019 to December 2020, 708 newly reported HIV-1 cases in Baoshan City were included in this study. After amplification and sequencing, 466 *gag*, 480 *pol*, and 470 *env* sequences were obtained, which were used for phylogenetic analysis (S2–S4 Figs). Based on at least two gene regions from one sample, 497 samples were successfully genotyped. There was no significant difference between the constituents of the 497 participants and those of the all participants (S1 Table).

Of the 497 individuals, 86.9% (432/497) were from China, and 13.1% (65/497) were from Myanmar. Among the five counties of Baoshan Prefecture, 78.3% (389/497) cases were reported in Longyang County and Tengchong County. There were 63.0% (313/497) and 37.0% (184/497) males and females, respectively. The average age was 40.6±13.2 years old. In total, 77.3% (384/497) were Han ethnicity; 22.7% (113/497) were Achang, Bai, Bulang, Dai, Hani, Hui, Jingpo, Lisu, Wa, Yao, Yi, and Zhuang nationalities. Regarding education level, 10.1% (50/497) were illiteracy; individuals who received primary school education, secondary education and high school education and above accounted for 39.0% (194/497), 38.2% (190/497) and 12.7% (63/497), respectively. Farmer was the main occupation (82.65%, 411/497). Heterosexual transmission was the dominant transmission route, accounting for 93.8% (466/497). The other routes (6.2%, 31/497) included homosexual transmission and intravenous drug use transmission. Among the 497 individuals, CD4^+^ T lymphocyte counts were available for 440, with proportions of CD4^+^ T lymphocytes counts < 200 cells/μl, 200–499 cells/μl and ≥500 cells/μl being 38.2% (168/440), 51.8% (228/440) and 10.0% (44/440), respectively.

### HIV-1 genotypes detected among the study participants

Among the 497 samples, 19 HIV-1 genotypes were found, including two subtypes, 16 CRFs and discrete URFs. The highest proportion was URFs (30.2%, 150/497), followed by CRF08_BC (18.9%, 94/497), CRF01_AE (12.3%, 61/497), CRF07_BC (9.3%, 46/497), CRF118_BC (7.4%, 37/497), CRF64_BC (6.2%, 31/497), Subtype C (6.0%, 30/497), Subtype B (2.8%, 14/497), CRF57_BC (1.2%, 6/497), CRF86_BC (1.2%, 6/497), CRF55_01B (1.0%, 5/497), CRF96_cpx (0.8%, 4/497), CRF62_BC (0.6%, 3/497), CRF100_01C(0.4%, 2/497), CRF101_01B(0.4%, 2/497), CRF65_cpx(0.4%, 2/497), CRF85_BC (0.4%, 2/497), CRF78_cpx (0.2%, 1/497) and CRF87_cpx(0.2%, 1/497). Among URFs, the most common recombinant form was BC recombinants (67.3%, 101/150); others included CRF01_AE/BC (15.3%, 23/150), CRF01_AE/C (10.7%, 16/150), CRF01_AE/B (4.0%, 6/150), CRF01_AE/CRF08_BC (1.3%, 2/150), and CRF07_BC/CRF08_BC (1.3%, 2/150).

### Spatial distribution of HIV-1 genotypes

**Fig 1:**
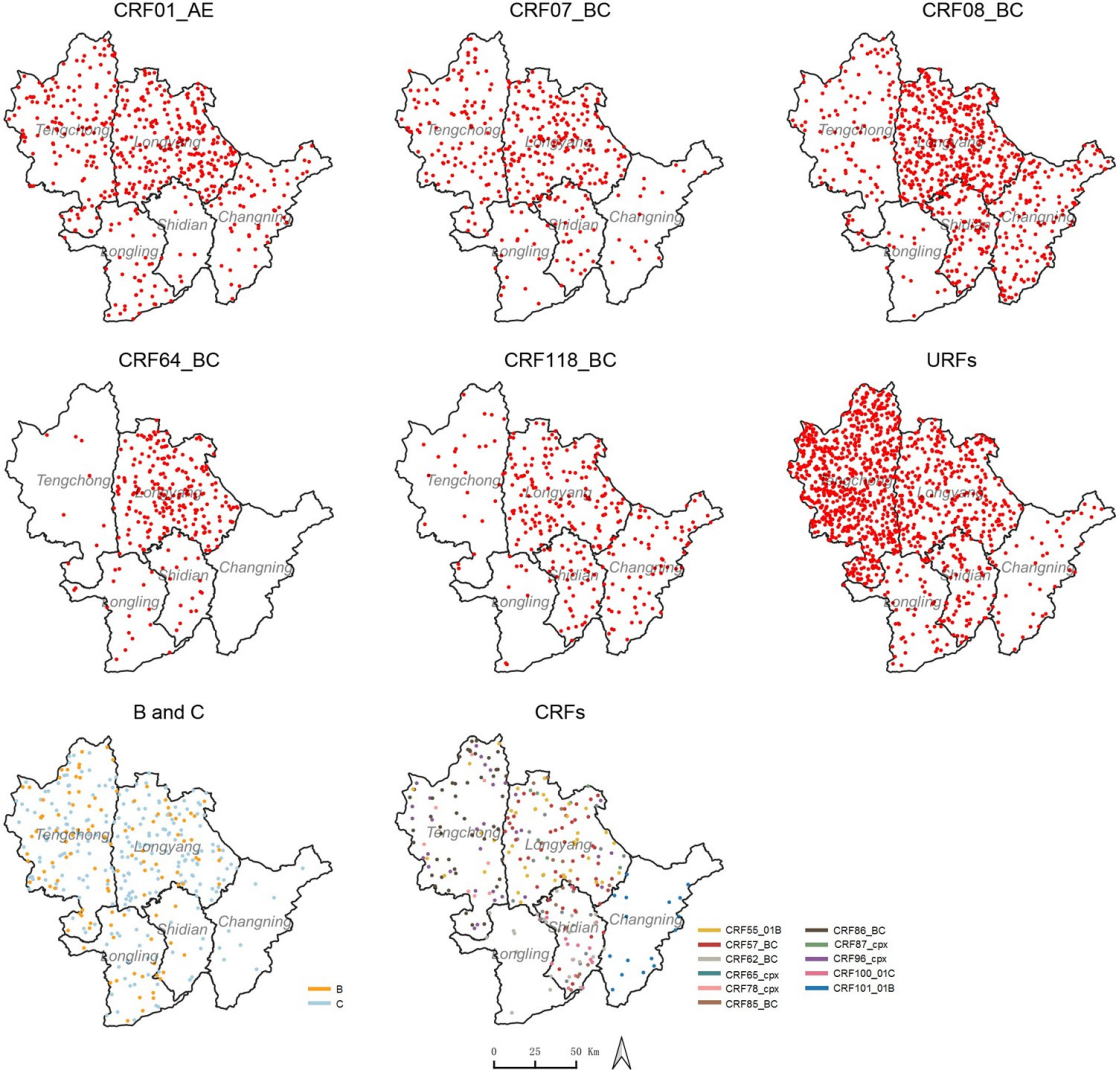
Spatial distribution of HIV-1 genotypes in Baoshan Prefecture. Dot density maps for CRF01_AE, CRF07_BC, CRF08_BC, CRF64_BC, CRF118_BC, URFs, Subtypes B and C, and CRFs. One dot represents 0.025% of the total cases genotyped. The shapefile of China was downloaded from the GADM database (www.gadm.org), version 3.4, April 2018, from which the shapefile of Baoshan Prefecture of Yunnan Province was extracted using Quantum GIS.

As illustrated in Fig 1, CRF01_AE, CRF07_BC, URFs and subtype C were mainly distributed in the two northern counties, Longyang and Tengchong. A higher prevalence of CRF64_BC was observed in Longyang. CRF08_BC and CRF118_BC were mainly found in the three eastern counties, Longyang, Shidian and Changning. Other CRFs were distributed sporadically in some counties.

### Demographic characteristics of HIV-1 genotypes

Characteristics associated with the distribution of the main HIV-1 genotypes were analyzed by univariate (S2 Table) and multivariate logistic regression (Table 1). Individuals aged <30 years (odds ratio (OR) (95% confidence interval (CI)): 4.397 (1.695, 11.404) and of Han ethnicity (OR (95% CI): 5.766 (1.894, 17.554)) were more likely to be infected with CRF01_AE. The proportion of CRF07_BC was higher in the subpopulation with an education level of junior middle school and above. The proportion of CRF08_BC was higher in the farmers (OR (95% CI): 2.296 (1.092, 4.828)) and those of Han ethnicity (OR (95% CI): 2.388 (1.188, 4.800)). CRF118_BC and CRF64_BC were more likely to be identified in the subpopulation aged ≥40 years and ≥50 years, respectively. URFs were more likely identified in Burmese individuals (OR (95% CI): 8.042 (3.463, 18.675)), intravenous drug users (OR (95% CI): 6.542 (1.730, 24.739)) and those younger than 50 years old.

**Table 1:** Demographic characteristics associated with HIV-1 genotypes.

			Total	Subject with given HIV-1 genotype	Proportion	Multivariate analysis	
						*p*	OR (95% CI)
CRF01_AE							
	Age					0.024*	
		≥50	132	8	6.1%	-	1.000
		40–49	118	16	13.6%	0.046*	2.496 (1.017, 6.129)
		30–39	126	15	11.9%	0.085*	2.271 (0.893, 5.771)
		<30	121	22	18.2%	0.002*	4.397 (1.695, 11.404)
	Race/ethnicity						
		Other	113	4	3.5%	-	1.000
		Han	384	57	14.8%	0.002*	5.766 (1.894, 17.554)
	Education					0.876	
		Illiteracy	50	4	8.0%	-	1.000
		Primary school	194	15	7.7%	0.764	0.832 (0.251, 2.759)
		Junior middle school	190	29	15.3%	0.877	1.098 (0.338, 3.561)
		Senior middle school and above	63	13	20.6%	0.873	0.892 (0.222, 3.592)
	Occupation						
		Farmer	411	42	10.2%	-	1.000
		Other	86	19	22.1%	0.093	1.847 (0.903, 3.779)
	Infection Route					0.609	
		Heterosexual contact	466	55	11.8%	-	1.000
		Homosexual contact	16	6	37.5%	0.319	1.852 (0.551, 6.228)
		Intravenous drug injection	15	0	0.0%	0.998	0(0,.)
CRF07_BC							
	Nationality						
		Non-Chinese	65	1	1.5%	-	1.000
		Chinese	432	45	10.4%	0.304	2.923 (0.378, 22.615)
	Gender						
		Female	184	11	6.0%	-	1.000
		Male	313	35	11.2%	0.360	1.416 (0.673, 2.98)
	Education					0.009*	
		Primary school	194	7	3.6%	-	1.000
		Junior middle school	190	22	11.6%	0.017*	2.981 (1.219, 7.290)
		Senior middle school and above	63	17	27.0%	0.001*	6.454 (2.204, 18.900)
		Illiteracy	50	0	0.0%	0.997	-
	Occupation						
		Farmer	411	29	7.1%	-	1.000
		Other	86	17	19.8%	0.410	1.380 (0.642, 2.970)
	Infection Route					0.829	
		Heterosexual contact	466	40	8.6%	-	1.000
		Homosexual contact	16	5	31.3%	0.582	1.409 (0.415, 4.780)
		Intravenous drug injection	15	1	6.7%	0.822	0.778 (0.087, 6.920)
CRF08_BC							
	Age					0.243	
		<30	121	14	11.6%	-	1.000
		30–39	126	25	19.8%	0.255	1.527 (0.737, 3.162)
		40–49	118	21	17.8%	0.668	1.181 (0.551, 2.531)
		≥50	132	34	25.8%	0.075	1.909 (0.936, 3.891)
	Race/ethnicity						
		Other	113	11	9.7%	-	1.000
		Han	384	83	21.6%	0.015*	2.388 (1.188, 4.800)
	Occupation						
		Other	86	9	10.5%	-	1.000
		Farmer	411	85	20.7%	0.028*	2.296 (1.092, 4.828)
CRF64_BC							
	Age					0.001*	
		<30	121	4	3.3%	-	1.000
		30–39	126	5	4.0%	0.781	1.209 (0.317, 4.612)
		40–49	118	3	2.5%	0.727	0.763 (0.167, 3.485)
		≥50	132	19	14.4%	0.005*	4.918 (1.623, 14.905)
CRF118_BC							
	Age					0.021*	
		<30	121	2	1.7%	-	1.000
		30–39	126	7	5.6%	0.123	3.500 (0.712,17.195)
		40–49	118	12	10.2%	0.014*	6.736 (1.474, 30.787)
		≥50	132	16	12.1%	0.006*	8.207 (1.846, 36.490)
URFs							
	Nationality						
		Chinese	432	97	22.5%	-	1.000
		Non-Chinese	65	53	81.5%	<0.001*	8.042 (3.463, 18.675)
	Gender						
		Male	313	86	27.5%	-	1.000
		Female	184	64	34.8%	0.211	1.348 (0.845, 2.150)
	Age					0.015*	
		≥50	132	19	14.4%	-	1.000
		40–49	118	35	29.7%	0.005*	2.568 (1.337, 4.930)
		30–39	126	44	34.9%	0.003*	2.780 (1.402, 5.512)
		<30	121	52	43.0%	0.038*	2.275 (1.048, 4.935)
	Race/ethnicity						
		Han	384	87	22.7%	-	1.000
		Other	113	63	55.8%	0.203	1.486 (0.808, 2.733)
	Education					0.253	
		Senior middle school and above	63	9	14.3%	-	1.000
		Junior middle school	190	48	25.3%	0.151	1.929 (0.788, 4.723)
		Primary school	194	66	34.0%	0.083	2.343 (0.894, 6.144)
		Illiteracy	50	27	54.0%	0.050	3.176 (1.002, 10.073)
	Occupation						
		Other	86	16	18.6%	-	1.000
		Farmer	411	134	32.6%	0.349	1.398 (0.694, 2.819)
	Infection Route					0.019*	
		Heterosexual contact	429	136	31.7%	-	1.000
		Homosexual contact	16	3	18.8%	0.491	1.653 (0.395, 6.911)
		Intravenous drug injection	15	11	73.3%	0.006*	6.542 (1.730, 24.739)

*: significant difference

### HIV-1 molecular network detected in the local area

**Fig 2:**
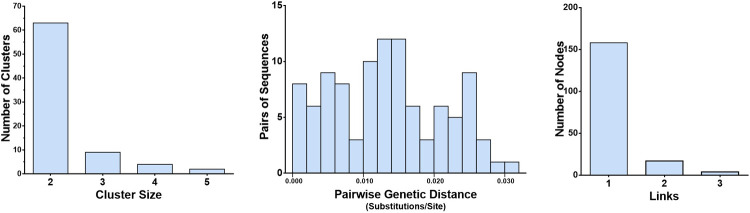
Characteristics of the HIV-1 molecular network in Baoshan Prefecture. A, Distribution of genetic transmission clusters by cluster size. B, Distribution of sequence pairs by genetic distance. C, Distribution of nodes in clusters by link.

The 480 *pol* sequences obtained were used to constructed an HIV-1 molecular network, of which 179 sequences (37.3%) grouped into 78 clusters. The cluster sizes ranged from two to five, and clusters sized two accounted for 83.8% (65/78). The median pairwise genetic distance was 0.0134 substitutions/site, and the interquartile range was 0.0066 to 0.0195 substitutions/site (Fig 2).

The associated demographic factors of the individuals clustered in the HIV-1 molecular network were further analyzed, including regional category, sex, age, ethnicity, marital status, education level, occupation and infection routes (Table 2). The regional category was the only factor with statistical significance in multivariate analysis (*p* = 0.014). Baoshan Prefecture individuals were more likely to cluster in the network than those from Myanmar (OR (95% CI): 2.464 (1.305, 4.651). The proportion of individuals from other cities in Yunnan Province and other provinces clustering in the network was equivalent to that of the individuals from Myanmar.

**Table 2:** Associated demographic factors of the individuals clustering in the HIV-1 molecular network.

Factors		Total	Cases detected in HIV-1 molecular clusters (%)	Univariate analysis		Multivariate analysis	
				*p*	OR (95% CI)	*p*	OR (95% CI)
Regional category				0.006		0.014*	
	Myanmar	65	15 (23.1%)	-	1.000	-	1.000
	Other Provinces	23	5 (21.7%)	0.895	0.926 (0.294, 2.914)	0.838	1.130 (0.349, 3.656)
	Other cities in Yunnan Province	31	8 (25.8%)	0.770	1.159 (0.431, 3.120)	0.587	1.322 (0.483, 3.621)
	Baoshan Prefecture	361	151 (41.8%)	0.005	2.397 (1.297, 4.428)	0.005*	2.464 (1.305, 4.651)
Sex							
	Male	304	101 (33.2%)	-	1.000	-	1.000
	Female	176	78 (44.3%)	0.016	1.600 (1.093, 2.342)	0.105	1.399 (0.933, 2.099)
Age				0.385			
	≤29	116	38 (32.8%)	-	1.000		
	30–39	119	48 (40.3%)	0.229	1.388 (0.814, 2.366)		
	40–49	115	39 (33.9%)	0.852	1.053 (0.609, 1.821)		
	≥50	130	54 (41.5%)	0.156	1.458 (0.866, 2.457)		
Race/ethnicity							
	Han	369	142 (38.5%)	-	1.000		
	Other	111	37 (33.3%)	0.326	0.799 (0.511, 1.250)		
Marital Status				0.016		0.430	
	Unmarried	136	37 (27.2%)	-	1.000	-	1.000
	Married	237	96 (40.5%)	0.010	1.822 (1.152, 2.880)	0.204	1.371 (0.842, 2.230)
	Divorced/Widowed	107	46 (43%)	0.011	2.018 (1.179, 3.454)	0.318	1.340 (0.754, 2.382)
Education				0.505			
	Senior middle school and above	63	19 (30.2%)	-	1.000		
	Junior middle school	176	66 (37.5%)	0.297	1.389 (0.749, 2.579)		
	Primary school	191	77 (40.3%)	0.151	1.564 (0.849, 2.881)		
	Illiteracy	50	17 (34%)	0.664	1.193 (0.539, 2.642)		
Occupation							
	Farmer	391	153 (39.1%)	-	1.000	-	1.000
	Other	89	26 (29.2%)	0.082	0.642 (0.389, 1.058)	0.277	0.747 (0.441, 1.265)
Infection Route				0.097		0.306	
	Heterosexual contact	449	173 (38.5%)	-	1.000	-	1.000
	Homosexual contact	17	2 (11.8%)	0.041	0.213 (0.048, 0.942)	0.125	0.300 (0.065, 1.396)
	Intravenous drug injection	14	4 (28.6%)	0.454	0.638 (0.197, 2.066)	0.861	0.898 (0.268, 3.007)

*: significant difference

### Transmission characteristics revealed by the HIV-1 molecular network

**Fig 3:**
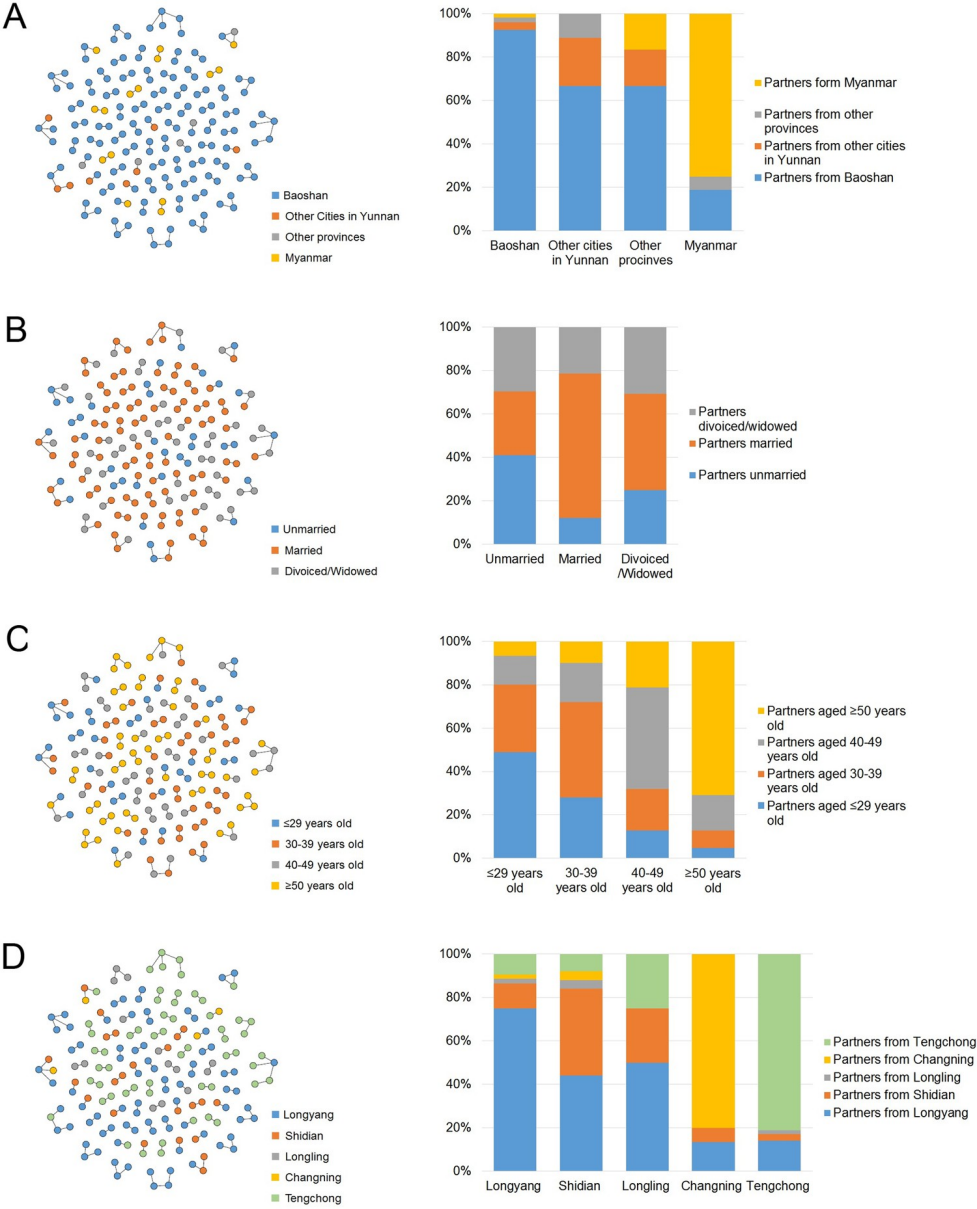
Relationship of the linked individuals in the HIV-1 molecular network in Baoshan Prefecture. A, Left panel: HIV-1 molecular clusters coded by regional category. Right panel: constitution of linked individuals stratified by regional category. B, Left panel: HIV-1 molecular clusters coded by marital status. Right panel: constitution of linked individuals stratified by marital status. C, Left panel: HIV-1 molecular clusters coded by age. Right panel: constitution of linked individuals stratified by age. D, Left panel: HIV-1 molecular clusters coded by counties where cases were reported. Right panel: constitution of linked individuals stratified by counties where cases were reported.

The relationship of the linked individuals was further analyzed (Fig 3). When stratified by the regional category of individuals in the network, the regional category of their linked individuals showed a significant difference (χ^2^ = 70.944, *p*<0.001). Among those linked with individuals from Baoshan Prefecture, other cities of Yunnan and other provinces, the proportions of Baoshan local individuals were 92.5%, 71% and 75%, respectively; 75.0% of those linked with the individuals from Myanmar were Burmese. When stratified by the marital status of individuals in the network, the marital status of linked individuals also differed significantly (χ^2^ = 23.199, *P*<0.001), the proportion of married individuals was higher among those linked with married individuals. When grouped by the age of individuals in the network, the age distribution of linked individuals in each group was significantly different (χ^2^ = 106.850, *P*<0.001); most people in each age group were linked to those in the same age range.

For the individuals reported in the different counties, the distribution of their linked individuals by county differed significantly (χ^2^ = 179.560, *P*<0.001), which suggested that the patterns of the crossing-counties transmission were different. Crossing-county transmission was detected in all five counties. However, 70–80% links in Longyang, Tengchong and Changning were intracounty; Over 50% of links in Shidian, were crossing-county transmission; and 100% of links in Longlin were crossing-county transmission.

### Dynamic transmission in the HIV-1 molecular network

**Fig 4:**
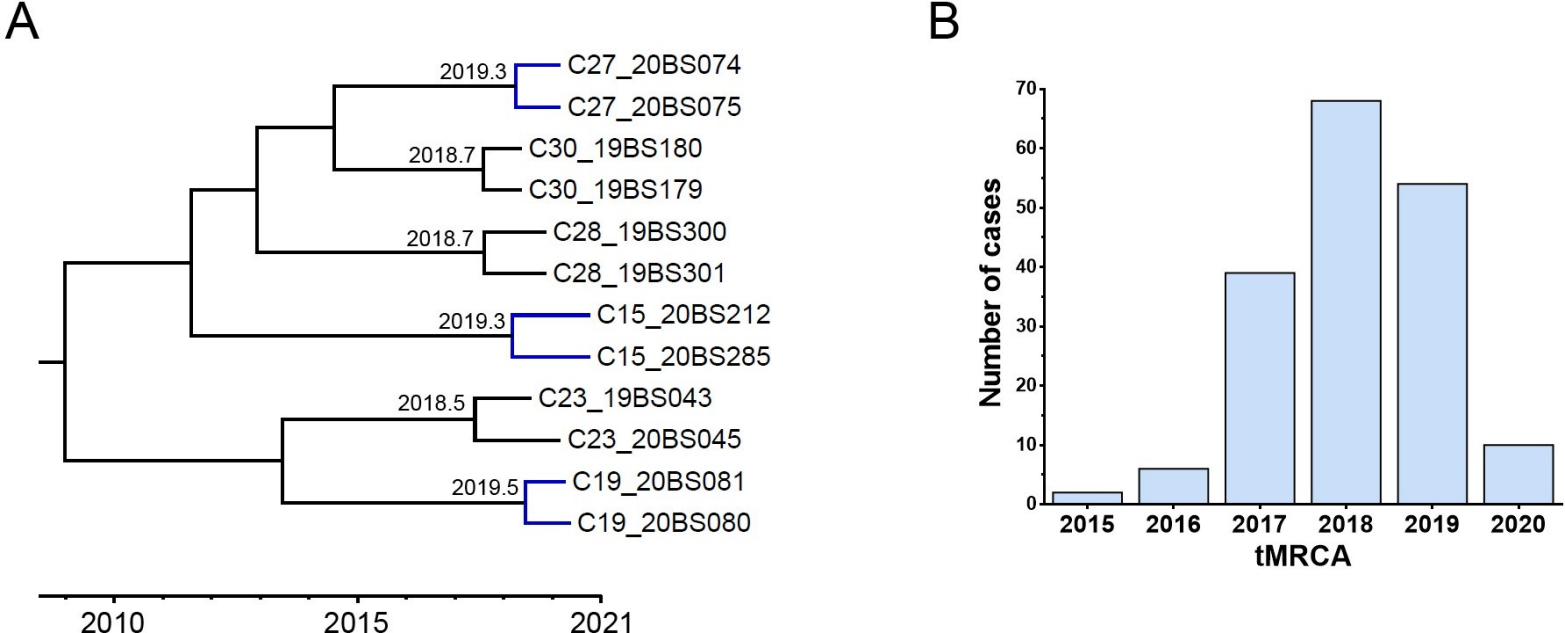
Distribution of tMRCA in the HIV-1 molecular network. A, Example inference of recent infection events. In the MCC tree, the tMRCA of the blue clades was post-2019, and these clades were associated with recent infection events. B, Distribution of tMRCA in the HIV-1 molecular network.

The time of the most recent common ancestor (tMRCA) of the clades within HIV-1 molecular clusters was estimated by Bayesian MCMC analysis, and it can be considered as the time when the transmission event occurred (Fig 4A). Nevertheless, whether either the descendant was the source or recipient partner with incident HIV it cannot be determined [10]. Among the clades in the molecular network, the median of tMRCA was 2018.7, and the range was 2015.1–2020.7 (Fig 4B).

In this study, a clade whose tMRCA was post-2019 was considered a recent infection event. Accordingly, 35.8% (64/179) of cases in the network were recent infections. The demographic characteristics of individuals associated with recent infection events were further analyzed (Table 3), and multivariate logistic analysis showed higher proportions of recent infections among nonfarmers and the youths (aged ≤29 years).

**Table 3:** Demographic characteristics of individuals associated recent infection events in the HIV-1 molecular network.

Factors		Total	Cases detected in HIV-1 molecular clusters (%)	Univariate analysis		Multivariate analysis	
				p	OR (95% CI)	p	OR (95% CI)
Regional category				0.322			
	Baoshan Prefecture	151	51	-	1.000		
	Other cities in Yunnan Province	8	3	0.829	1.176 (0.270, 5.120)		
	Other Provinces	5	4	0.069	7.843 (0.854, 72.006)		
	Myanmar	15	6	0.629	1.307 (0.441, 3.875)		
Sex							
	Male	101	33	-	1.000		
	Female	78	31	0.328	1.359 (0.735, 2.515)		
Age				0.075		0.211	
	≥50	54	11	-	1.000	-	1.000
	40–49	39	15	0.082	2.240 (0.903, 5.556)	0.130	2.038 (0.811, 5.120)
	30–39	48	20	0.032	2.560 (1.084, 6.045)	0.103	2.091 (0.862, 5.073)
	≤29	38	18	0.016	3.046 (1.226, 7.564)	0.050	2.546 (1.002, 6.468)
Race/ethnicity							
	Han	142	51	-	1.000		
	Other	37	13	0.930	0.967 (0.453, 2.061)		
Marital Status				0.556			
	Unmarried	37	16	-	1.000		
	Married	96	33	0.343	0.688 (0.317, 1.492)		
	Divorced/Widowed	46	15	0.321	0.635 (0.259, 1.556)		
Education				0.128			
	Senior middle school and above	19	11	-	1.000		
	Junior middle school	66	25	0.125	0.443 (0.157, 1.252)		
	Primary school	77	24	0.035	0.329 (0.118, 0.923)		
	Illiteracy	17	4	0.042	0.224 (0.053, 0.948)		
Occupation							
	Farmer	153	48	-	1.000	-	1.000
	Other	26	16	0.004	3.500 (1.480, 8.277)	0.019*	2.893 (1.195, 7.006)
Infection Route				0.926			
	Heterosexual contact	173	63	-	1.000		
	Homosexual contact	2	1	0.695	1.746 (0.107, 28.400)		
	Intravenous drug injection	4	0	0.999			

*: significant difference

## Discussion

To better understand the characteristics of HIV-1 transmission in western Yunnan Province, we performed a cross-sectional HIV-1 molecular epidemiological investigation in Baoshan Prefecture, one of the border prefectures in Yunnan Province., An HIV-1 molecular network was constructed and analyzed using HIV-1 sequence data.

HIV-1 genetics has become diverse in western Yunnan Province. In this study, 19 HIV-1 genotypes were found, and the proportion of URFs was far higher than that found previously [24, 30]. Among the 16 CRFs detected in this study, CRF01_AE, CRF07_BC and CRF08_BC were found or identified in the early stage of the HIV-1 epidemic in Yunnan Province [24, 30]. The other 13 CRFs are novel: 11 were originally identified in Yunnan Province, including CRF118_BC, CRF64_BC, CRF57_BC, CRF86_BC, CRF96_cpx, CRF62_BC, CRF100_01C, CRF101_01B, CRF65_cpx, CRF78_cpx and CRF87_cpx; CRF55_01 and CRF85_BC were first identified in Guangdong Province and Sichuan Province [31, 32].

The distribution of HIV-1 genotypes was different between Chinese and Burmese individuals. All 19 HIV-1 genotypes were found in Chinese individuals, whereas only five were found in Burmese individuals. Furthermore, multivariate logistic analysis showed that URFs were more likely to be detected in Burmese individuals. As reported in a previous study, extensive and complex HIV-1 recombination has occurred in northern Myanmar [33]. In Dehong, another prefecture bordering Myanmar, URFs were also the predominant strain [34], among which some CRFs were identified. A similar situation was observed in Baoshan, which suggests that the further studies are necessary, including the phylogeographic analysis and virulence analysis of recombinant strains.

CRF08_BC and CRF07_BC ranked the first and second, respectively, in recent infections in Yunnan Province [30]. In this study, the proportion of CRF08_BC was exceeded only by that of URFs. CRF08_BC was only found in Chinese individuals and was more likely to be detected among farmers and the individuals of Han ethnicity. The proportion of CRF07_BC was higher in the subpopulation with an education level of junior middle school and higher. Furthermore, CRF07_BC and CRF08_BC exhibited different spatial distribution patterns. These results suggests that these two CRFs have different transmission patterns.

CRF01_AE was first detected in FSWs who returned to Yunnan from Thailand in 1994 [14]. In the early 2000s, 85.4% of CRF01_AE infections were acquired via heterosexual transmission [35]. Among recent infections in Yunnan, the proportion of CRF01_AE decreased from 27.7% in 2009 to 11.4% in 2015 [24, 36]. Compared with CRF07_BC, CRF01_AE shows rapid disease progression, and low propagation dynamics [37]. A decrease in CRF01_AE and an increase in CRF07_BC were also observed in MSM [26, 38]. CRF01_AE was previously concentrated in the western region and gradually spread to the central region [24, 36]. In this study, CRF01_AE remained the primary strain in Baoshan Prefecture and was mainly transmitted among young persons (<30 years old) and the Han population.

CRF64_BC was first identified in a neighboring prefectures of Baoshan: Dehong [23], and a concentrated prevalence of CRF64_BC was first found in China in this study. The proportion of CRF64_BC was higher in the subpopulation aged >50 years. Although CRF118_BC was recently identified in Baoshan [20], it accounted for 7.4% and was widely distributed in all five counties. These results indicate that these two CRFs have been circulating for a long time in the local area.

Because 78.3% (389/497) of cases were reported in Longyang and Tengchong, the predominant HIV-1 genotypes were mainly distributed in these two counties or in one of these two counties, including CRF01_AE, CRF07_BC, CRF08_BC, CRF64_BC and URFs. Of Burmese cases, 84.6% (55/65) were reported in Tengchong; as URFs were more likely to be identified in Burmese individuals, the density of URFs in Tengchong was also higher than those in the other four counties.

In the last few years, HIV molecular networks have been gradually employed to monitor HIV dynamic transmission and guide targeted intervention. In this study, we attempted to use this approach to explore factors related to HIV transmission in the local area. For the cross-section of 2019–2020, 179 individuals grouped into 78 clusters, and the percentage of access to the network was 37.3%. Most clusters consisted of two individuals, and the largest clusters consisted of only five, suggesting that the overall epidemic to be in a sporadic state without obvious aggregation. The wide distribution of genetic distances may be related to the diversity of HIV-1 genetics in the local area, which indicates that it is not appropriate to use a uniform genetic distance as the criterion for molecular network construction.

Compared with Burmese, individuals from other cities in Yunnan and those from other provinces, Baoshan natives were more likely to be detected in the networks; thus, the network was localized. Among individuals linked with Chinese, including Baoshan natives, individuals from other cities in Yunnan and those from other provinces, 70%-90% were Baoshan natives in the molecular network. Moreover, 75% of those linked with Burmese were Burmese; however, 25% were Chinese, which indicates that transmission between Chinese and Burmese exists. Cross-border transmission mediated by Burmese had also been reported in Dehong [25]. Due to complicated HIV-1 genetics and the high prevalence of transmitted drug resistance among Burmese individuals [39], influx of Burmese might promote the complexity of HIV-1 genetics and challenge treatment in the border area.

The relationship among individuals in the molecular clusters also revealed specific transmission characteristics. Married individuals were more likely to be linked to married individuals. One possibility is that sexual behavior among those who are married is more complex, reflecting a changing concept of marriage. Another possibility is transmission between couples. Among 480 individuals with *pol* sequence detection, 31 had HIV-1-positive spouses or fixed sexual partners. This suggests that spouse notification and intervention for serodiscordant couples are important. In terms of age, individuals of the same age tended to have more connections, yet trans-age transmission was common in all age groups.

With the development of the economy and transportation, transregional transmission of HIV has become commonplace in China. In this study, we found that cases reported in different counties were linked with those reported in the other three to four counties. In Longyang, Tengchong and Changning, intracounty transmission accounted for the main proportion, and health education and behavioral intervention should be strengthened for the residents of these three counties. Longyang district is the seat of the prefectural government and is also centrally located. Cases reported in the other four counties were linked to those reported in Longyang district. Hence, AIDS prevention and control in Longyang district has a radiation effect. For Shidian and Longlin, cross-county transmission appeared to be predominant, and intervention in floating population should be considered in these two counties.

Incident infection, also called as recent infection, promotes to the growth of HIV molecular clusters, which should be prioritized [10]. Furthermore, we applied phylogenetic analysis to investigate the dynamic characteristics of the HIV-1 molecular network, focusing on the recent transmission events, and the time of transmission events was estimated based on the tMRCA of each clade in the network. Those clades with a tMRCA in the cross section of 2019–2020 were considered to be recent infections. Among the 179 individuals in the networks, 64 were involved in recent infection events. The proportion of recent infections was higher among nonfarmers, including some business service personnel. The proportion of recent infections was also higher among individuals aged ≤29 years, despite on significant difference, which suggests that recent infections more frequently occurred in the sexually active population. In general, intervention for these clusters will contribute to a decrease in future HIV incidence, including active anti-retroviral therapy. Moreover, tracing investigation for these clusters might facilitate identifying the population infected with HIV or at risk [7, 11].

### Conclusions

In this study, based on a cross-sectional HIV-1 molecular epidemiological investigation, we carried out HIV-1 molecular network analysis in western Yunnan. To our knowledge, the variety of HIV-1 genotypes detected in the current study exceeds those in the similar reports in Yunnan Province and in China. The diversity of HIV-1 genetics indicates complexity of HIV-1 transmission in the China-Myanmar border area. In addition, HIV-1 molecular network analysis revealed transmission characteristics that could not be obtained from conventional analysis. The findings will contribute to direct the transmission-reduction interventions, and our efforts will promote the development and application of an HIV-1 molecular surveillance network.

## Supporting information

S1 Fig Evaluation of the effect of the genetic distance threshold on cluster identification. A, Number of clusters changing with an increasing of genetic distance threshold. The node support threshold was ≥90%. B, Number of clustered sequences changing with an increase of genetic distance threshold. The node support threshold was ≥90%.
(PDF)

S2 Fig Neighbor-joining phylogenetic tree of the partial *gag* gene. The scale bar indicates 5% nucleotide sequence divergence. Values on the branches represent the percentage of 1000 bootstrap replicates.
(PDF)

S3 Fig Neighbor-joining phylogenetic tree of the partial *pol* gene. The scale bar indicates 5% nucleotide sequence divergence. Values on the branches represent the percentage of 1000 bootstrap replicates.
(PDF)

S4 Fig Neighbor-joining phylogenetic tree of the partial *env* gene. The scale bar indicates 20% nucleotide sequence divergence. Values on the branches represent the percentage of 1000 bootstrap replicates.
(PDF)

S1 Table The primers and procedures used for amplification and sequencing of HIV-1 gene fragments. (PDF)

S2 Table The constituents of the subjects successfully genotyped. (PDF)

S3 Table Demographic characteristics associated with HIV-1 genotypes (univariate analysis). (PDF)
