# Supplementary material for: Using molecular network analysis to explore the characteristics of HIV-1 transmission in a China-Myanmar border area
Source: PLoS One. 2022 May 6;17(5):e0268143. doi: 10.1371/journal.pone.0268143 (PMC9075624; doi:10.1371/journal.pone.0268143)
Supplement: S1 Fig — A, Number of clusters changing with an increasing of genetic distance threshold. The node support threshold was ≥90%. B, Number of clustered sequences changing with an increase of genetic distance threshold. The node support threshold was ≥90%. (PDF) [file pone.0268143.s001.pdf]

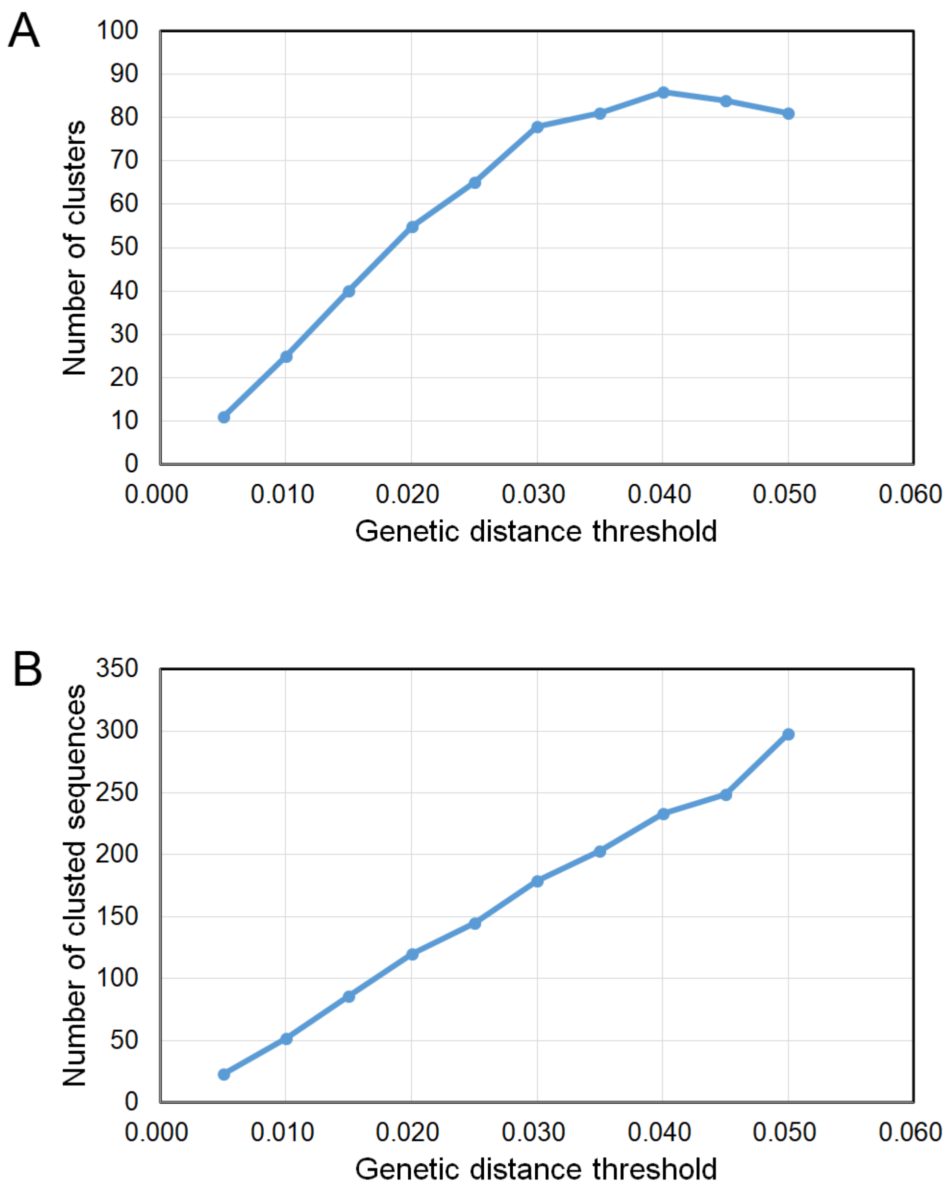

S1 Fig. Evaluation of the effect of the genetic distance threshold on cluster identification. A, Number of clusters changing with an increasing of genetic distance threshold. The node support threshold was ≥90%. B, Number of clustered sequences changing with an increase of genetic distance threshold. The node support threshold was ≥90%.
