# Supplementary material for: Using molecular network analysis to explore the characteristics of HIV-1 transmission in a China-Myanmar border area
Source: PLoS One. 2022 May 6;17(5):e0268143. doi: 10.1371/journal.pone.0268143 (PMC9075624; doi:10.1371/journal.pone.0268143)
Supplement: S4 Fig — The scale bar indicates 20% nucleotide sequence divergence. Values on the branches represent the percentage of 1000 bootstrap replicates. (PDF) [file pone.0268143.s004.pdf]

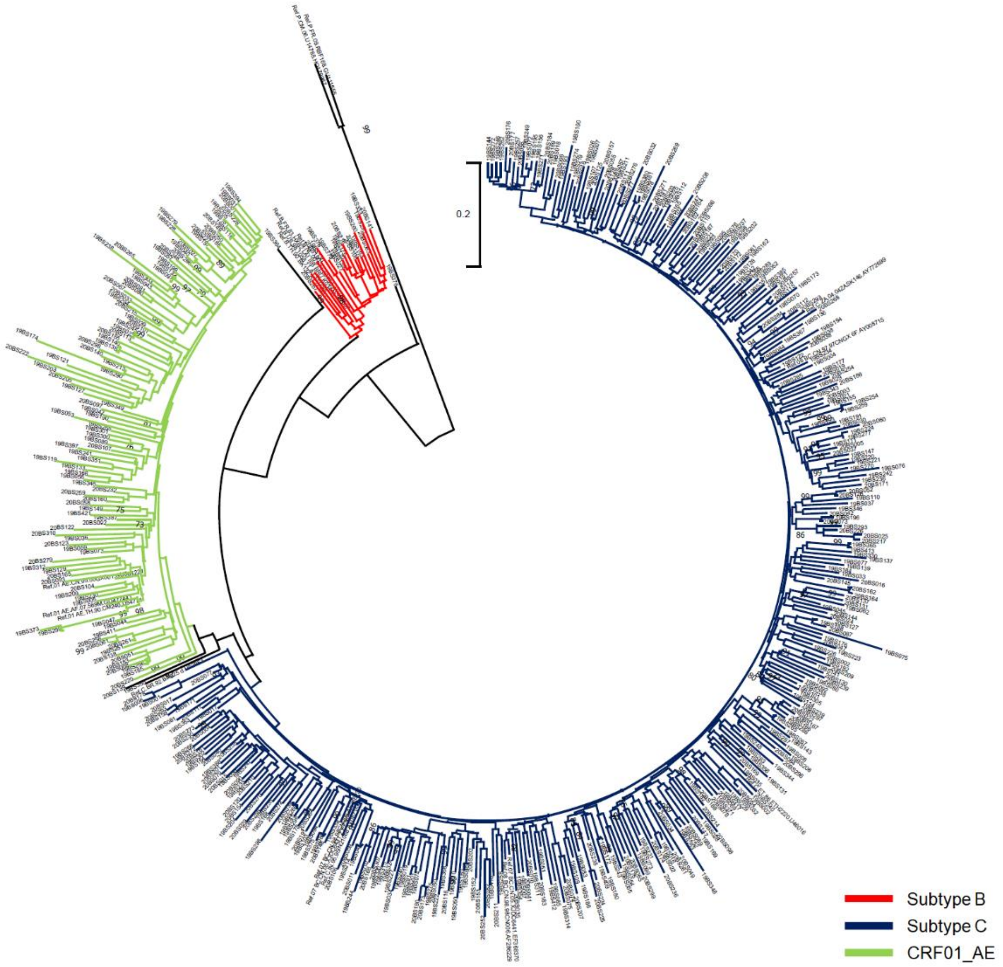

S4 Fig. Neighbor-joining phylogenetic tree of the partial *env* gene. The scale bar indicates 20% nucleotide sequence divergence. Values on the branches represent the percentage of 1000 bootstrap replicates.
