# Supplementary material for: Using molecular network analysis to explore the characteristics of HIV-1 transmission in a China-Myanmar border area
Source: PLoS One. 2022 May 6;17(5):e0268143. doi: 10.1371/journal.pone.0268143 (PMC9075624; doi:10.1371/journal.pone.0268143)
Supplement: S1 Table — (PDF) [file pone.0268143.s005.pdf]

S1 Table. The primers and procedures used for amplification and sequencing of HIV-1 gene fragments.

Region	Nested PCR	Primers	Cycling conditions	Sequencing primers
gag	1st PCR	GAG-L (5'- TCGACGCAGGACTCGGCTTGC -3')<br>GAG-E2 (5'- TCCAACAGCCCTTTTTCCTAGG -3')	50℃ for 30 min；94℃ for 5 min, 55℃ for 1 min，72℃ for 2 min；94℃ for 30 s，55℃ for 45 s，72℃ for 1 min 30 s，30 cycles；72℃ for 10 min.	GUX<br>GDX
	2nd PCR	GUX (5'-AGGAGAGAGATGGGTGCGAGAGCGTC-3')<br>GDX (5'- GGCTAGTTCCTCCTACTCCCTGACAT-3')	94℃ for 2 min, 55℃ for 1 min，72℃ for 1 min 30 s；94℃ for 30 s，55℃ for 45 s，72℃ for 1 min 30 s，30 cycles；72℃ for 10 min	
pol	1st PCR	MAW26 (5'-TTGGAAATGTGGAAAGGAAGGAC-3')<br>RT21 (5'-CTGTATTTCTGCTATTAAGTCTTTTGATGGG-3')	50℃ for 30 min；94℃ for 5 min；94℃ for 30 s，55℃ for 30 s，72℃ for 2 min 30 s，30 cycles；72℃ for 10 min.	PROS3 (5'-GCCAACAGCCCCACCA-3')<br>RTAS (5'-CTCAGATTGGTTGCAC-3')<br>RTB (5'-CCTAGTATAAACAATGAGACAC-3')<br>PROC1S (5'-GCTGGGTGTGGTATTCC-3')<br>RT20S3 (5'-GTTCTAGCTCTGCTTC-3')
	2nd PCR	PRO-1 (5'-CAGAGCCAACAGCCCCACCA-3')<br>RT20 (5¢-CTGCCAGTTCTAGCTCTGCTTC-3')	94℃ for 5 min；94℃ for 30 s，63℃ for 30 s，72℃ 2 min 30 s，30 cycles；72℃ for 10 min	
env	1st PCR	44F (5'-ACAGTRCARTGYACACATGG-3')<br>35R (5'-CACTTCTCCAATTGTCCITCA-3')	50℃ for 30 min；94℃ for 2 min, 50℃ for 1 min，72℃ for 4 min；94℃ for 30 s，55℃ for 30 s，72℃ for 2 min，30 cycles；72℃ for 10 min	33F<br>48R
	2nd PCR	33F (5'- CTGTTIAATGGCAGICTAGC -3')<br>48R (5'- RATGGGAGGRGYATACAT -3')	95℃ for 2 min; 95℃ for 15 s，55℃ for 30 s，72℃ 1 min 15 s, 5 cycles；95℃ for 15 s，60℃ for 30 s，72℃ for 1 min，25 cycles; 72℃ for 10 min	
