# Supplementary material for: Using molecular network analysis to explore the characteristics of HIV-1 transmission in a China-Myanmar border area
Source: PLoS One. 2022 May 6;17(5):e0268143. doi: 10.1371/journal.pone.0268143 (PMC9075624; doi:10.1371/journal.pone.0268143)
Supplement: S2 Table — (PDF) [file pone.0268143.s006.pdf]

S2 Table. The constituents of the subjects successfully genotyped.

	Subjects	Subjects obtaining suptypes	χ2	*P*
**Total**	708	497		
**Nationality**			2.386	0.129
Chinese	606	432		
Non-Chinese	102	65		
**County**			7.080	0.131
Longyang	300	223		
Shidian	56	41		
Longlin	39	24		
Changning	70	43		
Tengchong	243	166		
**Sex**			0.011	0.932
Male	445	313		
Female	263	184		
**Age**			1.847	0.608
<30	163	121		
31-39	185	126		
41-49	168	118		
≥50	192	132		
**Race/ethnicity**			0.018	0.922
Han	548	384		
Other	160	113		
**Marital Status**			0.700	0.702
Unmarried	199	140		
Married	345	246		
Divoiced/Widowed	164	111		
**Education**			1.013	0.801
Senior middle school and above	85	63		
Junior middle school	275	190		
Primary school	279	194		
Illiteracy	69	50		
**Occupation**			0.006	1.000
Farmer	586	411		
Other	122	86		
**Infection Route**			2.063	0.362
Heterosexual contact	660	466		
Homosexual contact	22	16		
Intravenous drug injection	26	15		
