# Supplementary material for: Using molecular network analysis to explore the characteristics of HIV-1 transmission in a China-Myanmar border area
Source: PLoS One. 2022 May 6;17(5):e0268143. doi: 10.1371/journal.pone.0268143 (PMC9075624; doi:10.1371/journal.pone.0268143)
Supplement: S3 Table — (PDF) [file pone.0268143.s007.pdf]

S3 Table. Demographic characteristics associated with HIV-1 genotypes (univariate analysis).

	Total	Subject with given HIV-1 genotype	Proportion	Univariate analysis	
				p	OR (95% CI)
CRF01_AE					
Nationality					
Non-Chinese	65	5	7.7%	-	1.000
Chinese	432	56	13.0%	0.233	1.787 (0.688, 4.642)
Sex					
Female	184	19	10.3%	-	1.000
Male	313	42	13.4%	0.312	1.346 (0.757, 2.393)
Age				0.040	
≥50	132	8	6.1%	-	1.000
41-49	118	16	13.6%	0.050	2.431 (1.000, 5.910)
31-39	126	15	11.9%	0.106	2.095 (0.855, 5.129)
<30	121	22	18.2%	0.004	3.444 (1.470, 8.069)
Race/ethnicity					
Other	113	4	3.5%	-	1.000
Han	384	57	14.8%	0.003	4.750 (1.684, 13.395)
Marital Status				0.197	
Unmarried	140	23	16.4%	-	1.000
Married	246	25	10.2%	0.075	0.575 (0.313, 1.058)
Divoiced/Widowed	111	13	11.7%	0.292	0.675 (0.325, 1.402)
Education				0.021	
Illiteracy	50	4	8.0%	-	1.000
Primary school	194	15	7.7%	0.950	0.964 (0.305, 3.042)
Junior middle school	190	29	15.3%	0.193	2.071 (0.693, 6.195)
Senior middle school and above	63	13	20.6%	0.071	2.990 (0.910, 9.829)
Occupation					
Farmer	411	42	10.2%	-	1.000
Other	86	19	22.1%	0.003	2.491 (1.366, 4.545)
Infection Route				0.020	
Heterosexual contact	466	55	11.8%	-	1.000
Homosexual contact	16	6	37.5%	0.005	4.484 (1.568, 12.819)
Intravenous drug injection	15	0	0.0%	0.999	-
CRF07_BC					
Nationality					
Non-Chinese	65	1	1.5%	-	1.000
Chinese	432	45	10.4%	0.049	7.442 (1.008, 54.944)
Sex					
Female	184	11	6.0%	-	1.000

Male	313	35	11.2%	0.057	1.980 (0.98, 4.002)
Age				0.186	
≥50	132	8	6.1%	-	1.000
41-49	118	13	11.0%	0.164	1.919 (0.766, 4.807)
31-39	126	9	7.1%	0.726	1.192 (0.445, 3.194)
<30	121	16	13.2%	0.058	2.362 (0.972, 5.738)
Race/ethnicity					
Other	113	6	5.3%	-	1.000
Han	384	40	10.4%	0.106	2.074 (0.856, 5.025)
Marital Status				0.386	
Divoiced/Widowed	111	7	6.3%	-	1.000
Married	246	23	9.3%	0.340	1.532 (0.637, 3.685)
Unmarried	140	16	11.4%	0.168	1.917 (0.760, 4.838)
Education				0.000	
Primary school	194	7	3.6%	-	1.000
Junior middle school	190	22	11.6%	0.005	3.498 (1.457, 8.398)
Senior middle school and above	63	17	27.0%	0.000	9.873 (3.866, 25.209)
Illiteracy	50	0	0.0%	0.997	-
Occupation					
Farmer	411	29	7.1%	-	1.000
Other	86	17	19.8%	0.000	3.245 (1.692, 6.225)
Infection Route				0.019	
Heterosexual contact	466	40	8.6%	-	1.000
Homosexual contact	16	5	31.3%	0.005	4.841 (1.602, 14.626)
Intravenous drug injection	15	1	6.7%	0.794	0.761 (0.097, 5.936)
CRF08_BC					
Nationality					
Chinese	432	94	21.8%	-	1.000
Non-Chinese	65	0	0.0%	0.997	-
Sex					
Male	313	62	19.8%	-	1.000
Female	184	32	17.4%	0.507	0.852 (0.532, 1.366)
Age				0.042	
<30	121	14	11.6%	-	1.000
31-39	126	25	19.8%	0.078	1.892 (0.931, 3.842)
41-49	118	21	17.8%	0.176	1.655 (0.797, 3.433)
≥50	132	34	25.8%	0.005	2.652 (1.343, 5.234)
Race/ethnicity					
Other	113	11	9.7%	-	1.000
Han	384	83	21.6%	0.006	2.557 (1.311, 4.986)
Marital Status				0.250	
Unmarried	140	20	14.3%	-	1.000
Married	246	52	21.1%	0.099	1.608 (0.915, 2.826)
Divoiced/Widowed	111	22	19.8%	0.245	1.483 (0.763, 2.883)

Education				0.223	
Illiteracy	50	4	8.0%	-	1.000
Primary school	194	41	21.1%	0.041	3.082 (1.048, 9.059)
Junior middle school	190	38	20.0%	0.056	2.875 (0.975, 8.481)
Senior middle school and above	63	11	17.5%	0.150	2.433 (0.725, 8.168)
Occupation					
Other	86	9	10.5%	-	1.000
Farmer	411	85	20.7%	0.031	2.231 (1.074, 4.631)
Infection Route				0.392	
Heterosexual contact	466	91	19.5%	-	1.000
Homosexual contact	16	1	6.3%	0.214	0.275 (0.036, 2.107)
Intravenous drug injection	15	2	13.3%	0.553	0.634 (0.141 ,2.859)
CRF64_BC					
Nationality					
Chinese	432	31	7.2%	-	1.000
Non-Chinese	65	0	0.0%	0.997	-
Sex					
Male	313	17	5.4%	-	1.000
Female	184	14	7.6%	0.335	1.434 (0.690, 2.982)
Age				0.001	
<30	121	4	3.3%	-	1.000
31-39	126	5	4.0%	0.781	1.209 (0.317, 4.612)
41-49	118	3	2.5%	0.727	0.763 (0.167, 3.485)
≥50	132	19	14.4%	0.005	4.918 (1.623, 14.905)
Race/ethnicity					
Han	384	27	7.0%	-	1.000
Other	113	4	3.5%	0.186	0.485 (0.166, 1.417)
Marital Status				0.216	
Unmarried	140	7	5.0%	-	1.000
Married	246	20	8.1%	0.251	1.681 (0.693, 4.082)
Divoiced/Widowed	111	4	3.6%	0.593	0.710 (0.203, 2.490)
Education				0.244	
Senior middle school and above	63	3	4.8%	-	1.000
Junior middle school	190	9	4.7%	0.994	0.994 (0.261 ,3.794)
Primary school	194	19	9.8%	0.225	2.171 (0.621, 7.598)
Illiteracy	50	0	0.0%	0.997	-
Occupation					
Farmer	411	26	6.3%	-	1.000
Other	86	5	5.8%	0.858	0.914 (0.341, 2.452)
Infection Route				1.000	
Heterosexual contact	466	31	6.7%	-	1.000
Homosexual contact	16	0	0.0%	0.999	-
Intravenous drug injection	15	0	0.0%	0.999	-
CRF118_BC					

Nationality					
Chinese	432	37	8.6%	-	1.000
Non-Chinese	65	0	0.0%	0.997	-
Gender					
Male	313	19	6.1%	-	1.000
Female	184	18	9.8%	0.131	1.678 (0.857, 3.286)
Sex				0.021	
<30	121	2	1.7%	-	1.000
31-39	126	7	5.6%	0.123	3.500 (0.712, 17.195)
41-49	118	12	10.2%	0.014	6.736 (1.474, 30.787)
≥50	132	16	12.1%	0.006	8.207 (1.846, 36.490)
Race/ethnicity					
Han	384	31	8.1%	-	1.000
Other	113	6	5.3%	0.329	0.639 (0.259, 1.571)
Marital Status				0.156	
Unmarried	140	6	4.3%	-	1.000
Married	246	19	7.7%	0.193	1.869 (0.728, 4.797)
Divoiced/Widowed	111	12	10.8%	0.054	2.707 (0.982, 7.461)
Education				0.393	
Senior middle school and above	63	2	3.2%	-	1.000
Junior middle school	190	14	7.4%	0.250	2.426 (0.536, 10.982)
Primary school	194	15	7.7%	0.221	2.556 (0.568, 11.498)
Illiteracy	50	6	12.0%	0.090	4.159 (0.801, 21.583)
Occupation					
Farmer	411	30	7.3%	-	1.000
Other	86	7	8.1%	0.787	1.125 (0.477, 2.653)
Infection Route				1.000	
Heterosexual contact	429	37	8.6%	-	1.000
Homosexual contact	16	0	0.0%	0.999	-
Intravenous drug injection	15	0	0.0%	0.999	-
URFs					
Nationality					
Chinese	432	97	22.5%	-	1.000
Non-Chinese	65	53	81.5%	0.000	15.253 (7.836, 29.692)
Sex					
Male	313	86	27.5%	-	1.000
Female	184	64	34.8%	0.087	1.408 (0.951, 2.083)
Age				0.000	
≥50	132	19	14.4%	-	1.000
41-49	118	35	29.7%	0.004	2.508 (1.341, 4.691)
31-39	126	44	34.9%	0.000	3.191 (1.736, 5.865)
<30	121	52	43.0%	0.000	4.482 (2.448, 8.206)
Race/ethnicity					
Han	384	87	22.7%	-	1.000

Other	113	63	55.8%	0.000	4.301 (2.766, 6.689)
Marital Status				0.238	
Unmarried	140	50	35.7%	-	1.000
Married	246	68	27.6%	0.099	0.688 (0.441, 1.073)
Divoiced/Widowed	111	32	28.8%	0.249	0.729 (0.426, 1.247)
Education				0.000	
Senior middle school and above	63	9	14.3%	-	1.000
Junior middle school	190	48	25.3%	0.075	2.028 (0.932, 4.415)
Primary school	194	66	34.0%	0.004	3.094 (1.439, 6.653)
Illiteracy	50	27	54.0%	0.000	7.043 (2.868, 17.298)
Occupation					
Other	86	16	18.6%	-	1.000
Farmer	411	134	32.6%	0.011	2.116 (1.184, 3.784)
Infection Routes				0.004	
Heterosexual contact	429	136	31.7%	-	1.000
Homosexual contact	16	3	18.8%	0.371	0.560 (0.157, 1.996)
Intravenous drug injection	15	11	73.3%	0.001	6.673 (2.088, 21.321)
